# A Practice-Proven Adaptive Case Management Approach for Innovative Health Care Services (Health Circuit): Cluster Randomized Clinical Pilot and Descriptive Observational Study

**DOI:** 10.2196/47672

**Published:** 2023-06-14

**Authors:** Carmen Herranz, Laura Martín-Moreno Banegas, Fernando Dana Muzzio, Antoni Siso-Almirall, Josep Roca, Isaac Cano

**Affiliations:** 1 Consorci d'Atenció Primaria de Salut Barcelona Esquerra Barcelona Spain; 2 Primary Healthcare Transversal Research Group Institut d'Investigacions Biomèdiques August Pi i Sunyer Barcelona Spain; 3 Institut Català de Salut Barcelona Spain; 4 Hospital Clínic of Barcelona Barcelona Spain; 5 Physiopathological Mechanisms of Respiratory Illnesses Group Institut d'Investigacions Biomèdiques August Pi i Sunyer Barcelona Spain; 6 Facultat de Medicina Universitat de Barcelona Barcelona Spain

**Keywords:** continuum of care management, innovative healthcare services, collaborative tools, digital health transformation, usability, acceptability, health care service, Health Circuit, health management, management, support, digital aid, aid, care, prototype, surgery, testing

## Abstract

**Background:**

Digital health tools may facilitate the continuity of care. Enhancement of digital aid is imperative to prevent information gaps or redundancies, as well as to facilitate support of flexible care plans.

**Objective:**

The study presents Health Circuit, an adaptive case management approach that empowers health care professionals and patients to implement personalized evidence-based interventions, thanks to dynamic communication channels and patient-centered service workflows; analyze the health care impact; and determine its usability and acceptability among health care professionals and patients.

**Methods:**

From September 2019 to March 2020, the health impact, usability (measured with the system usability scale; SUS), and acceptability (measured with the net promoter score; NPS) of an initial prototype of Health Circuit were tested in a cluster randomized clinical pilot (n=100) in patients with high risk for hospitalization (study 1). From July 2020 to July 2021, a premarket pilot study of usability (with the SUS) and acceptability (with the NPS) was conducted among 104 high-risk patients undergoing prehabilitation before major surgery (study 2).

**Results:**

In study 1, Health Circuit resulted in a reduction of emergency room visits (4/7, 13% vs 7/16, 44%), enhanced patients’ empowerment (*P*<.001) and showed good acceptability and usability scores (NPS: 31; SUS: 54/100). In study 2, the NPS was 40 and the SUS was 85/100. The acceptance rate was also high (mean score of 8.4/10).

**Conclusions:**

Health Circuit showed potential for health care value generation and good acceptability and usability despite being a prototype system, prompting the need for testing a completed system in real-world scenarios.

**Trial Registration:**

ClinicalTrials.gov NCT04056663; https://clinicaltrials.gov/ct2/show/NCT04056663

## Introduction

Worldwide, conventional disease-oriented approaches are being replaced by innovative, preventive, patient-centered health care services with digital health tools that support vertical (between the hospital and the community) and horizontal (between health and social services) integration. However, despite the broad consensus there are still significant challenges in ensuring care continuum across health organizations [1].

In many cases, cooperation among health care tiers and providers needs digital support for secure bidirectional and instantaneous communication to avoid information gaps or duplications and assist the care team in making shared decisions. Moreover, the nondeterministic nature of health care services requires a shift in focus from a set of prescriptive tasks to context-dependent, flexible, often-evolving care plans aimed at achieving optimal outcomes for each patient.

In 2001, adaptive case management (ACM) was introduced in the workflow management literature to support knowledge workers (eg, physicians, architects, and lawyers) [2] and unpredictable business processes that require flexibility. Because knowledge-based work is data-intensive, the ACM approach can foster more informed decisions and customize business processes as necessary [3,4]. The application of these ACM principles is essential for efficient digital health transformation. Improved end-user engagement, personalization to the evolving patient’s health status, and better communication and coordination between health care professionals are just some examples of the benefits of ACM when applied to innovative health services [5].

The current study presents the precommercial experience of co-design and prototyping of a digital health tool (Health Circuit) with an ACM approach during the period from 2019 to 2021. The process had 2 consecutive steps. In an initial phase, from 2019 to 2020, the feasibility of a secure bidirectional communication channel was tested for improving the management of complex chronic patients with high risk of hospitalization. In a second phase (2020-2021), ACM principles were incorporated to enable virtual sharing of care plans among all team members of an innovative prehabilitation service [6-8] that provided both home and community-based care to candidates for major abdominal surgeries.

While the first pilot study aimed to analyze the service’s potential with respect to management of unplanned events and patient self-management, both studies shared the common objective of assessing the Health Circuit approach with respect to its usability and acceptability as perceived by end users.

## Methods

### Context

It is of note that the precommercial experience reported in this study benefited from the combined input of all the stakeholders, including end users, from a health district integrated care area in the city of Barcelona (Área Integral de Salud Barcelona Esquerra; AISBE), which includes 520,000 citizens. All stakeholders have long experience in piloting digital support for enhanced management of chronic patients [9,10]. The project was part of the activities of the Catalan Open Innovation Hub on Digitally Enabled Integrated Care Services, which is 1 of the 4 original European Union Good Practices in the European Joint Action on Implementation of Digitally Enabled Integrated Person-Centered Care (JADECARE) [11].

Within this regional digital health transformation context, the first reported study for prevention of hospitalizations in complex chronic patients (2019-2020) focused on assessment of a prototype-level (technology readiness level [TRL] 5) [12] secure bidirectional communication channel with a twofold purpose: (1) facilitating management of unplanned events through direct access to a single case manager who could eventually trigger a shared decision-making process with other health professionals (primary care, specialists, or both) and (2) empowering patients’ self-management with the aim of increasing self-efficacy through shared videos and educational material.

The second reported study (2020-2021) focused on assessing the acceptability and usability of a pilot version of Health Circuit (TRL 7) for enhancing patient adherence to the prehabilitation program at the Hospital Clinic of Barcelona (HCB) [13,14]. In recent years, prehabilitation has emerged as a health-value intervention to reduce postoperative morbidity and mortality in a variety of surgical populations [6]. In related work, we have previously demonstrated the efficacy [7] and potential effectiveness [8,15] of prehabilitation. It is of note that the impact of prehabilitation in real-world settings seems strongly associated with actionable areas, such as enhancing patients’ adherence and transferability to community settings, which requires digital support with an ACM approach [14] to enable virtual sharing of care plans among professionals across health tiers and prescription of nonpharmacological interventions to promote patient empowerment for self-management.

### Health Circuit Approach

The ACM approach of Health Circuit aims to empower professionals to define dynamic service workflows as patient-centered, evidence-based interventions with special input on combining pharmacological and nonpharmacological interventions. It can be viewed as a semiautomated and event-driven task manager for professionals and patients that enables them to perform certain activities in the context of the patients’ treatment.

**Figure 1:**
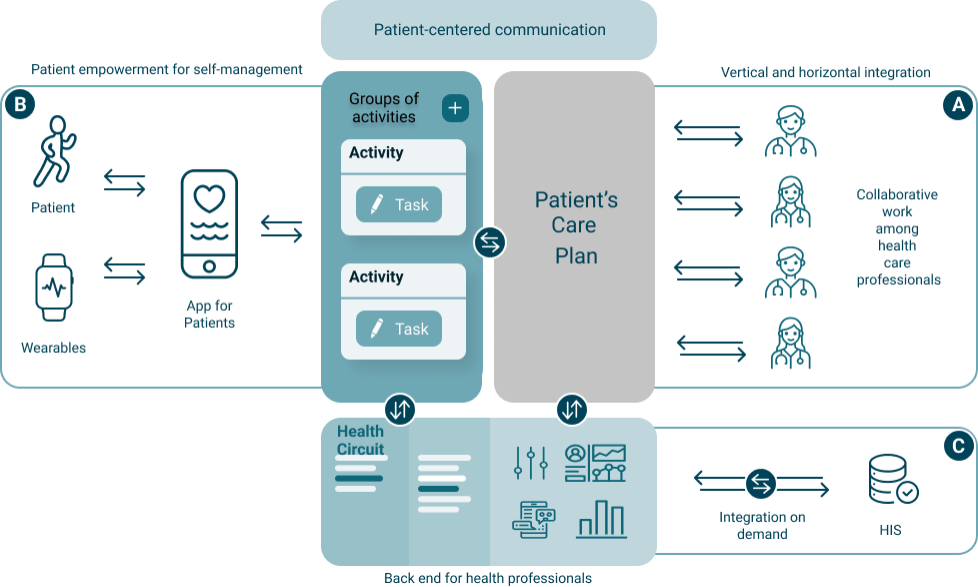
Schematic depiction of Health Circuit. (A) Health care professionals easily adapt and customize shared care plans over time, facilitating a connected experience for both the patient and the health care professional. (B) Patients participate in effective health coaching and self-management strategies, on any device, for a goal-oriented and personalized health plan to manage both expected and unexpected events. (C) Without requiring tight system integration, Health Circuit allows health care providers to progressively replace conventional disease-oriented approaches with a predictive, preventive, personalized, and participatory integrated care approach. HIS: health information system.

It is collaborative in the sense that health care professionals must share their knowledge and decisions with their peers (Figure 1).

### Main Features of Health Circuit

#### Case-Centered

The collaboration is bounded by the case, meaning that a group of professionals interact with each other and the patient in the context of the case.

#### Multichannel Communication

The communication channel is a tool for helping in decision-making. A secure chat between a number of peers starts with a particular issue in mind and, when resolved, leaves a summary of the decision and motivations for it, which then is translated into some action. It is also used to help patients solve doubts and issues with their treatment. This is the front-line support to collaborative work.

#### Multidisciplinary Shared Care Plan

The activities to be performed are related to different specialties and disciplines from various levels of care but are shared in a common care plan. A care plan represents the workflow of how a treatment is planned to be addressed. It has a layered structure with 3 levels of elements, comprising, from bottom to top, tasks, activities, and groups of activities. Tasks are what is ultimately actionable. They represent things like “the third session of physiotherapy at the hospital gym,” “filling in some questionnaires for the second time,” and “the fifth day of receiving nutritional tips.” Activities generate identical tasks over a period with a given recurrence. They can be started or ended automatically by linking them to events related to other activities. Groups of activities help enabling or disabling activities at a higher level (eg, baseline assessments; weekly follow-ups).

The structural dependencies between the above elements, in the sense of the care plan or service workflow that represents the treatment, translate into 2 main concepts: semiautomated processes, comprising activities that are dependent on or related to the outcome or completion of other activities; and overridability, that is, using structures that do not impose strict rules on professionals, so they can override any decision with proper motivation; this is registered into the system for accountability.

#### Event-Driven Architecture

Health Circuit is designed with an event-driven architecture in mind. This means that the internal structure of the care plan is based in events and actions (ie, a publish-subscribe software architecture pattern) that can be linked together. Events are anything that happens, from “the activity A started” or “the group of activities X ended” to “the weekly total step count passed the 80% threshold.” Actions are a limited set of operations that can be performed, like “start or end an activity or activity group” or “send a push, SMS, or email notification to a patient or professional.” More advanced scenarios will introduce actions that represent integration with third party systems, like “upload a report to the health information system.”

Such an event-driven architecture is scalable and open and allows introducing a visual workflow editor, so that health care professionals without technical skills can define and customize their own care plan templates.

#### Tailored Integration With Existing Corporate-Specific Health Information Systems

Delivery as a low-barrier corporate communication channel without requiring tight system integration will maximize adoption across the global health care information technology (IT) market. When required, Health Circuit can be easily integrated with site-specific health information systems by means of standards-based (eg, Health Level Seven Fast Health Interoperability Resources; HL7-FHIR) interoperability middleware, provider-specific web services, or application programming interfaces. This can be the case for the calendar of appointments, the agenda of health care professionals, admission and discharge reports, and authentication with corporate-specific centralized user management systems.

Compared to digital health solutions developed through a traditional consultancy process, Health Circuit allows multidisciplinary health care teams and patients to start collaborating right away via the multimedia communication channel, preparing the stakeholders to formalize the need for coordination over time based on a shared care plan representing a workflow template of how a treatment is agreed to be addressed. This means that health care professionals can face new cases reusing structured, evidence-based experience with previous cases without needing any special skills. Over time, health care professionals should be able to adapt the system to their own evidence-based care plans without needing the help of any IT specialist.

### Community-Based Management of Complex Chronic Patients With High Risk for Hospitalization

The protocol was designed as a cluster randomized controlled trial conducted in 3 primary care teams pertaining to the Consorci d’Atenció Primària de Salut Barcelona Esquerra (CAPSBE) primary care area of Barcelona, with an intervention to control ratio of 2:1 and a follow-up period of 3 months. Eligible patients (n=100) were selected from among 441 participants of the study [16] who had a referral primary care team in CAPSBE. Due to the nature of the intervention, neither the participants nor the investigators in direct contact with them were blinded. The doctors included were the reference doctors of each of the study patients and were not selected by the researchers. They all agreed to participate in the study, consent was given, and randomization was conducted.

The inclusion criterion was an adjusted morbidity group (AMG) score ≥3 [17,18]; this is a proxy of moderate to high risk based on multimorbidity criteria. AMG is an aggregative index that estimates the individual’s burden of disease from a disease-specific weighting obtained from a population-based statistical analysis based on mortality and the use of health services. Moreover, patients or caregivers in the intervention group were required to have a smartphone or tablet compatible with the Health Circuit prototype mobile app with an Internet connection. The exclusion criteria were physical or psychological problems preventing the use of Health Circuit and being unemployed.

The study run between September 2019 and March 2020. For both the intervention and the control group, sociodemographic data and digital literacy characteristics were collected at baseline, the Fantastic Questionnaire [19] and Elders Health Empowerment Scale (EHES) [20] were collected at baseline and at 3 months, and the patients’ and professionals’ satisfaction with and assessments of the usability of the Health Circuit prototype and the continuity of care were measured with the self-administered net promoter score (NPS) [21], the System Usability Scale (SUS) [22], and the Nijmegen Continuity Questionnaire [23], respectively. All data were collected face to face at the beginning and at the end of the study and was stored on the RedCap platform. Multimedia Appendix 1 provides details.

For the intervention group, a motivational interview was conducted at baseline with one case manager who aimed to co-design a personalized and comprehensive care plan and empower the patient for self-management of their conditions (Table 1). The nurse case manager carried out follow-up visits at 15 days, 1 month, and 2 months after the start of the study and were always available during the 3 months of the intervention when the patients reported any query or health problem through the app. Patients and professionals affiliated with the intervention primary care centers were instructed on the functionalities of the Health Circuit prototype (Multimedia Appendix 2).

In the control group, both patients and professionals followed standard of care procedures for regular and emergency visits, both by telephone and in person, depending on the center’s availability.

Results are presented as means (SD) or percentages, as indicated. Comparisons were made using the chi-square or Fisher exact test for categorical variables and the Student test (according to the distribution of the variables) for numerical variables.

**Table 1:** Sociodemographic baseline characteristics of 47 patients with high risk for hospitalization.

			Intervention (n=31)		Control (n=16)		*P* value^a^	
Age (years), mean (SD)			72.7 (13.7)		78.1 (8.8)		.11	
Male, n (%)			20 (64)		12 (75)		.52	
Higher educational level, n (%)			19 (61)		6 (38)		.25	
Widowed, n (%)			14 (45)		1 (6)		.004	
Living with family, n (%)			22 (71)		14 (88)		.25	
Retired, n (%)			20 (64)		15 (94)		.11	
**AMG^a^ score, n (%)**								.46
	Moderate risk (80th to 95th percentiles)	15 (48)		7 (44)				
	High risk (95th to 99th percentiles)	16 (52)		9 (56)				

^a^AMG: adjusted morbidity group, an index indicating multimorbidity and the complexity of the patient, expressed in percentiles [17,24].

### Prehabilitation of High-Risk Candidates for Major Surgical Procedures

A descriptive observational study was carried out including patients (n=104) who completed the prehabilitation program at HCB 8 between July 2020 and July 2021 and agreed to participate in the study. The study was offered to all patients included in the prehabilitation program on the dates described (n=213).

The inclusion criterion was having a smartphone or tablet compatible with the prototype mobile app (Prehab) with an Internet connection. The exclusion criteria were (1) physical or psychological problems precluding the use of the mobile app and (2) not having a carer.

The prototype version of Health Circuit tailored to the prehabilitation process (Prehab; TRL 7) allowed care team members to prescribe and monitor nonpharmacological interventions for patient self-management through a mobile app, which included (1) physical activity goals, (2) nutritional tips, (3) mindfulness exercises, and (4) patient-reported outcomes. Moreover, patients had access to a one-to-one chat with the case manager [25] for enhanced management of multimorbidity. Multimedia Appendix 3 provides details.

At the end of the prehabilitation program (4-6 weeks on average), the patients’ satisfaction with and assessments of the usability of the mobile app were collected with the self-administered NPS and SUS, respectively. Moreover, the patients’ use of the mobile app was evaluated according to (1) the number of chat interactions, (2) adherence to reporting of daily physical activity, (3) completion of follow-up questionnaires, and (4) access to the tailored education material on mindfulness, nutrition, and physical activity. Results are presented as means or percentages, as indicated.

In this study the back end for health care professionals was not assessed because the recruitment and follow-up of study participants was done with only one health care professional, a case manager physiotherapist.

### Ethics Approval

Study approval was obtained for the cluster randomized clinical pilot from the Ethics Committee for Clinical Research of the HCB (HCB/2018/0805), and the trial was registered at ClinicalTrials.gov (NCT04056663) on August 14, 2019. Patients read, understood, and filled out the informed consent form, which was signed before enrollment in the study. The Ethics Committee for Medical Research of HCB approved the premarket pilot study (HCB/2016/0883). Informed consent forms were understood, accepted, and signed by all the included subjects.

## Results

### Community-Based Management of Complex Chronic Patients With High Risk for Hospitalization

Two primary care units were randomly assigned as intervention units and 1 as a control unit. From an initial sample of 100 eligible patients, 41 did not meet the participation criteria. The Consolidated Standards of Reporting Trials (CONSORT) study flowchart is shown in Multimedia Appendix 4. The remaining 59 patients were assigned to one of the study arms according to the reference center. A total of 47 patients, 31 in the intervention arm and 16 controls, completed follow-up. The initial demographic characteristics are shown in Table 1.

Detailed information on the availability of smartphones (32/36, 88% in the intervention group and 13/18, 72% in the control group), digital baseline characteristics, baseline use of technology, and health information sources at the beginning of the study can be found in Multimedia Appendix 5.

During the study period, a total of 28 consultations, 12 clinical and 16 administrative, were done by 26 of 31 patients in the intervention group. In all cases, the events were solved in a timely manner. Offline chat was used in 78% (22/28) of the consultations and phone calls in the remaining 22% (6/28).

The intervention had positive clinical impacts in terms of less use of health care resources, increased patient empowerment (*P*<.001), and improved perception of continuity of care (*P*<.001). As depicted in Table 2, the intervention group had fewer hospital (0 vs 1) and primary care (4 vs 7) emergency room visits, a lower hospitalization rate (0 vs 2), less mortality (0 vs 2), and half the number of visits to primary care (27 vs 52) compared to the control group. However, these results were not statistically significant. The difference in Δ empowerment (pre-post) in the intervention group and the control group was 9 points (4.5 vs –4.56).

**Table 2:** Clinical and patient-reported outcomes during the study period.

Outcome variables	Intervention (n=31)	Control (n=16)	*P* value
Hospital emergency room visits, n	0	1	N/A^a^
Primary care emergency room visits, n (%)	4 (13)	7 (44)	N/A
Primary care visits, n (mean per patient)	27 (1.59)	52 (3.25)	N/A
Hospitalizations, n (%)	0 (0)	2 (13)	N/A
Mortality, n (%)	0 (0)	2 (13)	N/A
Baseline lifestyle [19], mean score (SD)	41.18 (3.53)	36.06 (4.36)	N/A
Δ Lifestyle (pre-post) [19], mean score (SD)	2.62 (4.49)	0.43 (4.15)	N/A
Baseline empowerment [20], mean score (SD)	36.46 (5.38)	28.44 (6.24)	<.001
Δ Empowerment (pre-post) [20], mean score (SD)	4.5 (5.23)	-4.56 (5.38)	<.001
Continuity of care [23], mean score (SD)	4.84 (2.40)	2.75 (1.26)	<.001

^a^N/A: not applicable.

### Usability and Acceptability

The patients reported good satisfaction and usability for the mobile app (NPS score 31 and SUS 68/100, respectively), as well as a high acceptance rate (mean score 7.8/10). However, the backend for health professionals had a neutral score in terms of satisfaction and usability (NPS score –80 and SUS 54/100, respectively) with an acceptance score averaging 5/10. Despite these results for the acceptability and usability of the backend, 75% (12/16) of the professionals participating in the study indicated that the remote consultations had good overall quality, 87% (14/16) reported that they would continue to use remote consultations as a support tool, and 69% (11/16) of the professionals considered that telemedicine can improve the state of patients’ health.

### Prehabilitation of High-Risk Candidates for Major Surgical Procedures

A total of 104 patients of 213 (mean age 69, SD 11 years, 67% men) that agreed to download the Prehab mobile app (TRL 7) [26,27] during the study period were included in the study.

The mobile app obtained good usability scores (NPS score 40 and SUS 85/100), as well as a high acceptance rate (mean score 8.4/10).

The patient’s primary use of the mobile app was to follow the prescription for daily physical activity, in 96% (n=100) of the cases. The weekly nutrition follow-up questionnaires were completed by 40% (n=42) of the patients; 37% (n=38) of study participants used the chat at least once to communicate with the prehabilitation team members (ie, physiotherapists and nutritionists). Only 21% (n=22) of the participants completed the daily mindfulness exercises. Educational tips on nutrition and physical activity were used by 33% (n=34) and 26% (n=27) of the study participants, respectively.

## Discussion

### Principal Results

We have presented the Health Circuit ACM approach to help health care professionals create patient-centered workflows combining both pharmacological and nonpharmacological interventions. It functions as a semiautomated task manager for professionals and patients, allowing them to perform certain activities as part of the patient’s action plan. This approach encourages collaboration and sharing of knowledge among health care professionals. We assessed the feasibility of the approach for two innovative health care services: (1) enhanced management of complex chronic patients with high risk of hospitalization through a secure bidirectional communication channel and (2) scalability of a prehabilitation service that was previously demonstrated to be efficient [7] and cost-effective [8,15], incorporating the virtual sharing of health plans that include nonpharmacological activities toward patient empowerment for self-management.

The pilot study on community-based management of complex chronic patients with high risk for hospitalization (with a TRL of 5) showed the potential of the approach to enhance management of unplanned health events, leading to less use of health care resources as well as improved patient empowerment for self-management and perception of continuity of care. The results are encouraging, especially when considering that both patients and health care professionals reported reasonably good usability and acceptability, despite the high mean age of the study participants (72.7 years) and the series of limitations that prototype-level digital health tools with a low TRL tend to have. Similarly, Health Circuit, which has a TRL of 7, was reported to have good usability and acceptability in the pilot study on prehabilitation of high-risk candidates for major surgical procedures, improving patient adherence to nonpharmacological treatment, especially in the case of daily physical activity.

### Strengths and Weaknesses

Despite the precommercial nature of the digital health tools supporting each of the 2 feasibility studies, reasonably good usability and acceptability results were obtained in real-world clinical settings. The positive results reinforce the need to pursue the development of a commercial product with potential for cost-effectiveness and profitability in real-world settings.

However, we must acknowledge several limiting factors of the studies, such as small sample sizes, the local health care settings, and the descriptive observational study design of study 2, which may preclude the generalization of the findings to other populations or health care settings.

Finally, although one of the limiting factors for adoption of value-based care services is still acceptability and usability among stakeholders, especially older people, the reported studies contribute to the growing body of evidence that indicates the positive effects of eHealth services on patient health outcomes [28-31] (eg, enhanced patient empowerment and adherence to care plans) at a lower cost.

### Adaptive Case Management

Change management is critical for ensuring effective digital health transformation, as it helps stakeholders to adapt to new processes and technologies, build support for change, and achieve the goals of the transformation. By taking an ACM approach, health care organizations can minimize disruption, ensure stakeholder buy-in, and improve the quality of care. Briefly, front-line support for flexible case-centered collaboration requires secure multichannel communication, whereas shared care plans can be introduced to multidisciplinary care teams in a stepwise manner, leading to a more standardized care protocol as the result of the effective adoption of organizational interoperability.

### Perspectives and Future Developments

The aim of Health Circuit is to facilitate large-scale adoption of value-based care services, such as the 2 innovative health care services considered in this study. Therefore, future actions should focus on easing the implementation of digitally enabled, integrated, person-centered care by means of a flexible approach to shared care processes and a holistic approach to health risk assessment.

In this vision, ACM processes dictate information needs, which is a great opportunity to decouple data from applications, enabling data to follow patient-centric care processes and helping to scale up innovations for value-based health care. Storing data with open standards, such as OpenEHR, allows data analysts to develop the secondary use of understanding cost-effectiveness within the context of a certain care plan. With this infostructure, AI experts can train automated decision support for health risk assessment. This can potentially also be done for health risk assessment to indicate the best care plan for a given type of patient. This minimizes certain risks, like mortality or unplanned hospital admissions.

### Conclusions

The Health Circuit approach to ACM demonstrated potential for value generation, as well as reasonably good usability and acceptability results among end users. The 2 pilot studies validated the feasibility of the approach to enable collaboration and knowledge-sharing among health care professionals from different levels of care and with patients.

Therefore, Health Circuit can play a crucial role for the adoption of patient-centered, integrated care by enabling health care professionals from different disciplines and organizations to make better-informed decisions about patient care, which can lead to personalized medicine based on health risk assessment that considers the needs and circumstances of each patient.

## Appendix

Multimedia Appendix 1 Net promoter score and System Usability Scale measures.

Multimedia Appendix 2 User manual: Community-based management of complex chronic patients with high risk for hospitalization.

Multimedia Appendix 3 User manual: Prehabilitation of high-risk candidates for major surgical procedures.

Multimedia Appendix 4 Flow diagram of Health Circuit.

Multimedia Appendix 5 Digital baseline characteristics in the intervention group and baseline use of technologies and health information sources.

Multimedia Appendix 6 CONSORT-eHEALTH checklist (V 1.6.1).

## Abbreviations

ACM: adaptive case management
AISBE: Área Integral de Salud Barcelona Esquerra
AMG: adjusted morbidity group
CAPSBE: Consorci d’Atenció Primària de Salut Barcelona Esquerra
EHES: Elders Health Empowerment Scale
HCB: Hospital Clinic of Barcelona
HIS: Health information system
HL7-FHIR: Health Level Seven Fast Health Interoperability Resources
IT: information technology
NPS: net promoter score
SUS: system usability scale
TRL: technology readiness level
