# Supplementary material for: A Practice-Proven Adaptive Case Management Approach for Innovative Health Care Services (Health Circuit): Cluster Randomized Clinical Pilot and Descriptive Observational Study
Source: J Med Internet Res. 2023 Jun 14;25:e47672. doi: 10.2196/47672 (PMC10337458; doi:10.2196/47672)
Supplement: Multimedia Appendix 1 [file jmir_v25i1e47672_app1.docx]

**MULTIMEDIA APPENDIX 1: NPS and SUS measures**

The Net Promoter Score is a known questionnaire used to assess satisfaction with a product, which includes a key question: “How likely is it that you would recommend our system to a family member or friend?”. Patients can give an answer ranging from 0 (“not at all likely”) to 10 (“extremely likely”). Individuals scoring a 9 or a 10 are called “promoters”, individuals scoring 7 or 8 are called “passives” (or neutrals) and individuals scoring 0 to 6 are labelled as “detractors”. The SUS was developed by John Brooke in 1986 and consists of a 10-item questionnaire scored on a 5-point Likert scale from 0 (strongly disagree) to 5 (strongly agree). The overall score is calculated from a sum of all item scores multiplied by 2.5 and can range from 0 to 100. A system or product that received score of 68 and above is considered to have good usability
