# Supplementary material for: A Practice-Proven Adaptive Case Management Approach for Innovative Health Care Services (Health Circuit): Cluster Randomized Clinical Pilot and Descriptive Observational Study
Source: J Med Internet Res. 2023 Jun 14;25:e47672. doi: 10.2196/47672 (PMC10337458; doi:10.2196/47672)
Supplement: Multimedia Appendix 2 [file jmir_v25i1e47672_app2.docx]

**MULTIMEDIA APPENDIX 2: User manual: Community-based management of complex chronic patients with high risk for hospitalization**

**• Language selection:** As a user, you can select between three languages: Spanish, Catalan and English.

**• Tutorial:** As a user, when I download the app, I can see a short tutorial of the main functionalities that the app offers .


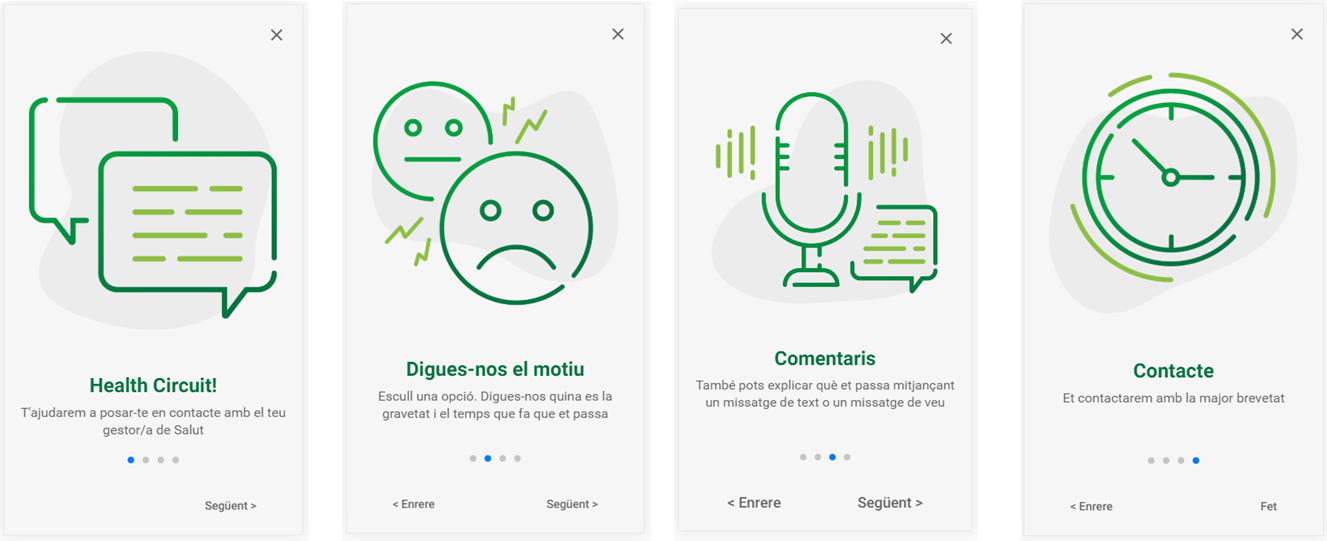


- **Login:** As a user I must access with an email and password provided by the clinic. In the HealthCircuit app, I can view the key to reduce the risk of entering an incorrect password.
  - Particularities: You are only asked to enter the username and password the first time you access 🡪The user must be aware that when logging in he/she consents to the data of the connection being saved (through consent)
    - As long as the user does not log out, he will be able to access Health Circuit without the need to enter a password.
    - If the user logs out, he must re-enter the username and password. In addition, while you are not connected to Health Circuit, you will not be able to receive communications from the clinic (messages or calls), you will only be able to see the messages received once you log back in as a user.
    - The user must be informed about:
      - The Hospital Clínic has a database with logins and passwords pseudonymized _
      - You only need to enter the login the first time you access the app. Therefore, in case of loss, theft, etc., it is recommended to notify the Clinic immediately.
      - Phase I: All the information sent is registered in Circuit. The user must be informed of the storage time of the conversations. To comply with GDPR, any data we save (conversations in circuit) must be based on the legitimacy of the treatment (Why we save it and the knowledge of the interested party about the treatment and retention of data).
      - Phase II: By incorporating the chat option, all the information sent through triage is recorded both in Circuit and on the user's device. To comply with GDPR, any data we save (conversations in circuit) must be based on the legitimacy of the treatment (Why we save it and the knowledge of the interested party about the treatment and retention of data).
    - If the user has forgotten the password, they can request a reminder by calling the Clinic.


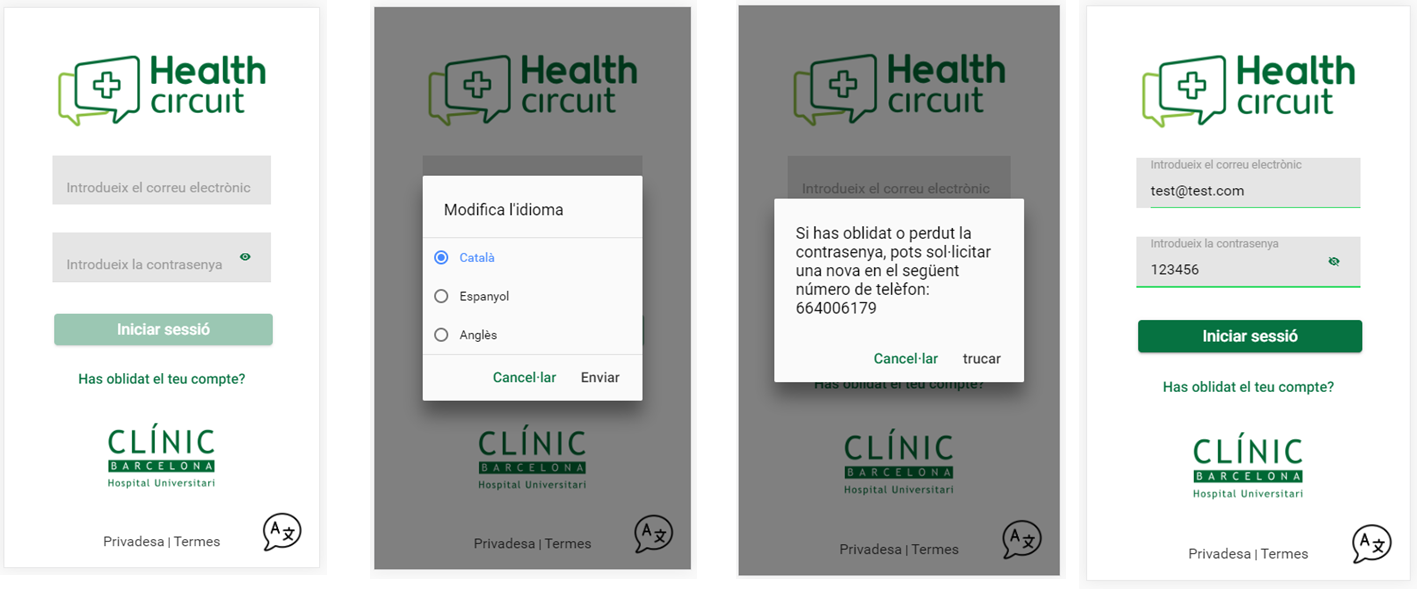


- **Triage:**
  - As a user, I can select from the following options: Type of inquiry: Pain, Shortness of breath, Fever, Other complaints or Administrative doubts *.
  - Symptom intensity **: Very intense, intense, neutral, mild or very mild.
  - Time since symptom onset **: Less than 1h, between 1h-12h, between 12h-24h, between 2-7 days, More than 1 week, More than 1 month.
  - Optional: The user is given the possibility to detail the reason for the query by recording an audio or sending a text message.

* For the "Administrative Doubts" option, the intensity or time since symptom onset screens are not generated. The user is redirected directly to the audio or text registration screen.

** Both sections are mandatory to enter to go to the next screen.


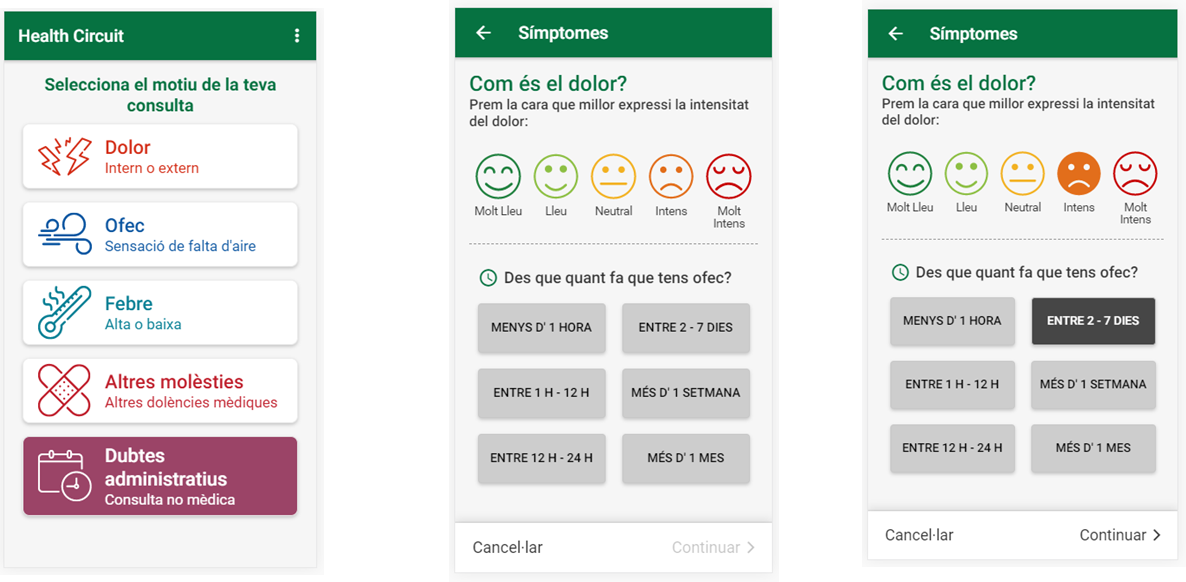


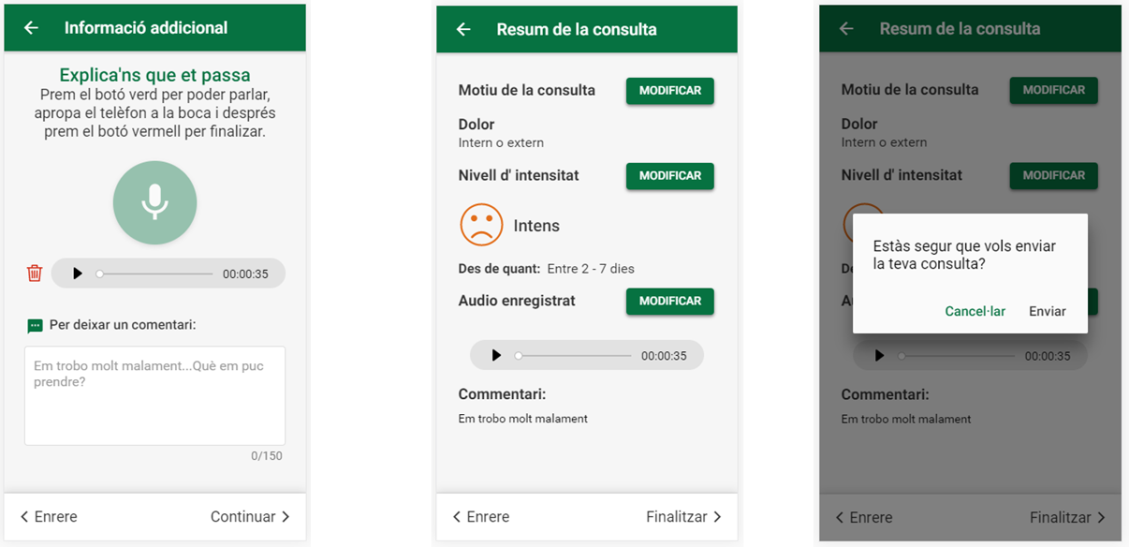


- **Chat:**
  - Once the triage has been completed and sent, the "Manager / a not available" screen will automatically appear: Your health manager / a will contact you within 3 hours at the most (from Monday to Friday from 08:00 a.m. to 08:00 p.m.). If you cannot wait, we recommend that you call 061 or go directly to your usual health center. Additionally, you can contact us again via chat enabled for any query related to the sent triage status. "


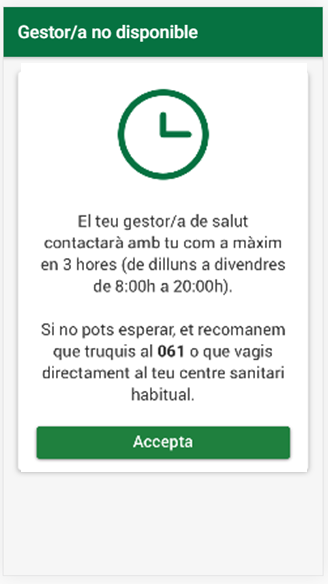


- - As a user, upon acceptance, I will automatically be redirected to the initial triage screen where I will have chat enabled. I will be able to access the chat icon and type any additional details about the submitted triage (Example: My condition has worsened). I will also be able to receive triage-related messages from the Clinic when the health manager is available, within a maximum of 3 hours from the sending of the triage by the user.
  - If I wish to create a new triage again, a message will appear when selecting any of the 5 options, stating " Are you sure you want to create a new triage? Creating a new triage means that the chat conversation associated with the last triage will be automatically removed. " You can select "Yes" or "Back"
  - If I select "Yes", the chat from the last triage will be deleted and a new one will be created when the triage submission is complete.
  - The Clinic, for its part, through Circuit, will not be able to reply to triages sent previously, it will only be able to reply about the last triage.
- **Push notifications:**
  - As a user, as long as I have not logged out of the application, I may receive notifications of incoming calls / video calls or incoming messages, even if the application is not open in the foreground.
  - If the user has logged out, notifications will not be received. You will only be able to view the messages received through the chat when you log in again.
    - Particularities:
      - The user, through the signed consent, must authorize receiving calls / messages in Push notifications. A settings section where you can activate / deactivate notifications is not included. Therefore, you must be aware that if you do not wish to receive push notifications, you must contact the Clinic.
- **The Clinic may contact the participant regardless of whether there is an event or not.**
  - At any time and regardless of whether or not a triage has been created, the Clinic can contact the user.
  - For this and with the aim of complying with GDPR, it is necessary for the user to consent and to know for what reason they may be contacted. You must include in the terms of privacy and in the consent to be signed by the user for which purpose he can be contacted and accept it.
