# Supplementary material for: A Practice-Proven Adaptive Case Management Approach for Innovative Health Care Services (Health Circuit): Cluster Randomized Clinical Pilot and Descriptive Observational Study
Source: J Med Internet Res. 2023 Jun 14;25:e47672. doi: 10.2196/47672 (PMC10337458; doi:10.2196/47672)
Supplement: Multimedia Appendix 3 [file jmir_v25i1e47672_app3.docx]

**MULTIMEDIA APPENDIX 3: User manual: Prehabilitation of high-risk candidates for major surgical procedures**


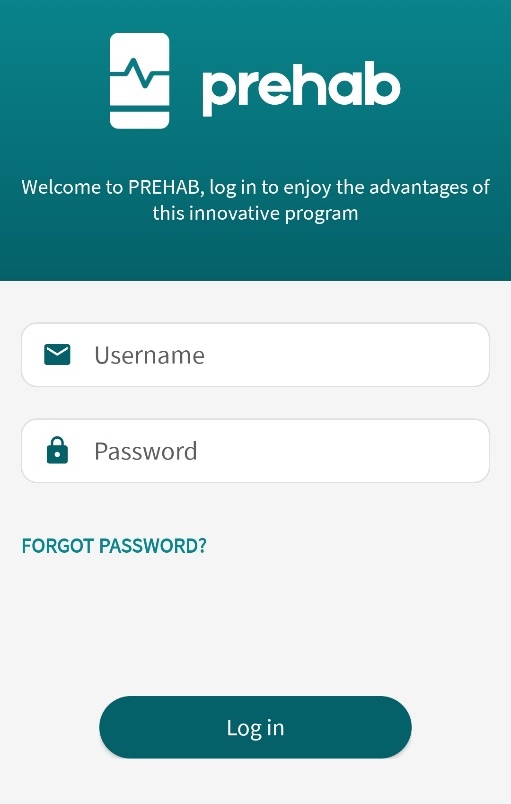


**Login**

To use Prehab, the app user’s credentials must be validated by following the instructions from the clinician.

You will need to enter your **username** and **password** for access.

On the same screen you’ll see the **“Forgot password?”** option, so it can be changed if you've forgotten it.

In addition, you can view the app’s **terms and conditions**.


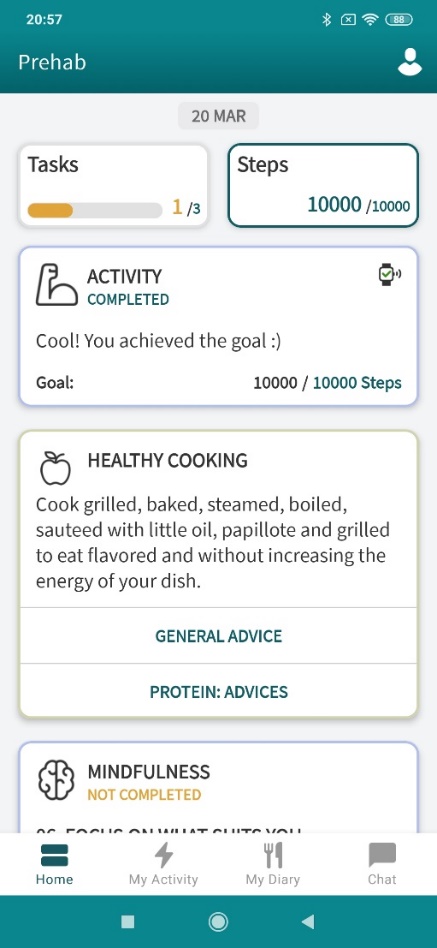
**Home screen**

This is the screen you’ll see when you start a session as a patient. It contains the following information:

The **Profile** icon is at the top; it can be used to view your user information or information about the connection with your activity wristband, and it also allows you to log out.

Beneath it, you’ll see two fields: the **Tasks** field shows the number of activities to be completed during the day, and the **Steps** field shows the number of steps to be taken.

The other fields refer to instructions received from clinical specialists. There are also fields with resources for the Prehab programme and a welcome video.

The lower menu shows the following options: **Home**, to return to the home screen; **My Activity**, where you can more closely monitor your physical activity; **My Diary**, where you can take and display photos of what you've eaten each day; and **Chat**, where you can start a chat with clinicians.

**
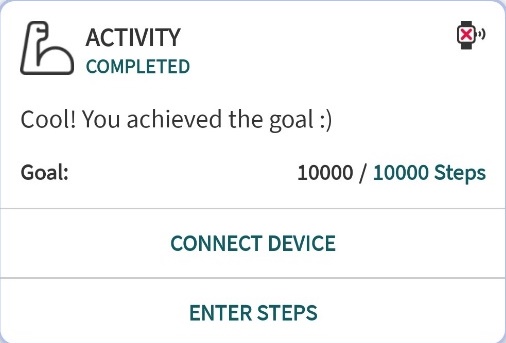
Activities**

The following activities may be visible for the patient:

**Physical activity:** This lets you know how many steps to take, and gives you the option to enter steps manually if you don’t have an activity wristband. You can also connect your device to measure your steps automatically.


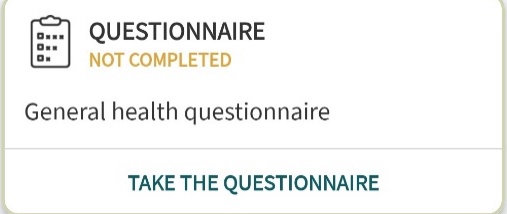


**Questionnaires:** The type of prescribed questionnaire is displayed, and you have the option to complete it. Once you've entered your answers, the status will change from “Not completed” to “Completed,” and you can view your responses.


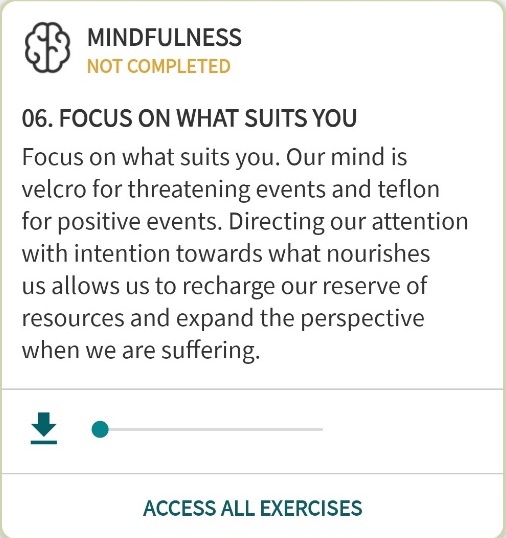


**Mindfulness:** An introductory text is displayed, and you’re given the option of completing the day’s proposed mindfulness exercise. You also have the option of viewing all the exercises in case you want to do more.


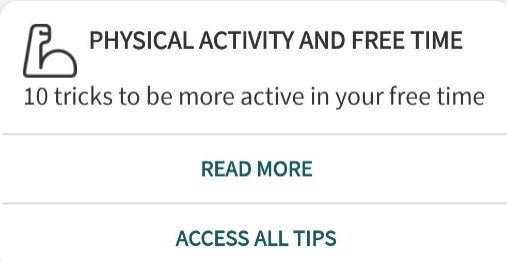


**Physical activity advice:** Each day a different piece of advice from the clinicians will be displayed. You’ll also be able to view all the advice if you want more information.


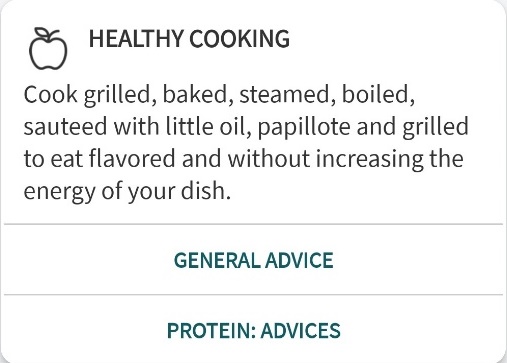


**Nutrition advice:** This section contains nutrition advice provided by the clinicians and will be a great help to patients. A different piece of advice will be posted each day.


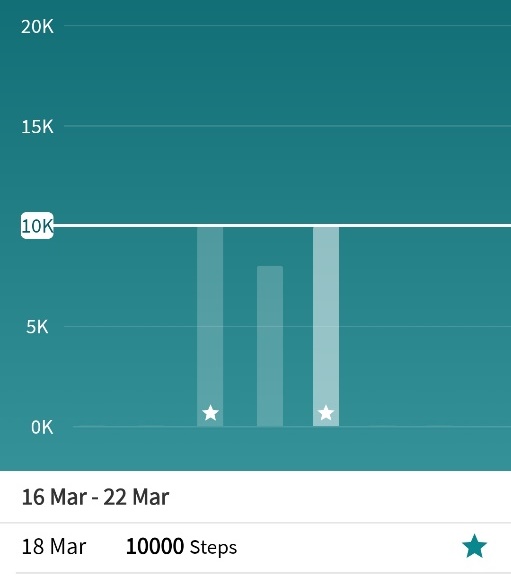


**My activity**

On this screen you can monitor your physical activity more closely with the aid of a bar graph showing the number of steps taken each day.

A white horizontal line shows your goal so you can easily see whether you've reached it.


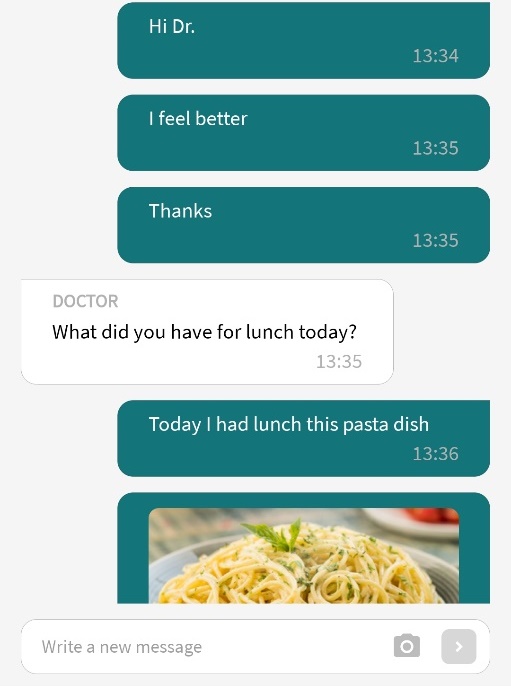


**Chat**

On the Chat screen you can quickly and easily exchange messages with clinicians. You can also send clinicians a photo stored on your mobile or a photo taken with the camera.

Clinicians can also send PDF documents, which will appear on the chat screen so the patient can read them.

**
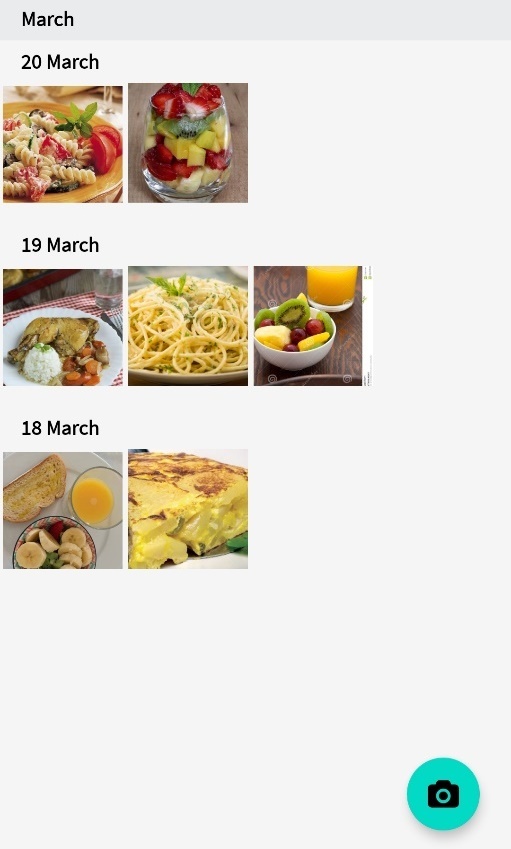
**

**Food diary**

This option appears only if the clinician has prescribed a food diary for the patient.

This screen allows patients to send photos of what they've eaten during the day so that the clinician can monitor their diet.

The various photos taken each day can also be viewed, with the most recent ones appearing first.
