# Supplementary material for: A Practice-Proven Adaptive Case Management Approach for Innovative Health Care Services (Health Circuit): Cluster Randomized Clinical Pilot and Descriptive Observational Study
Source: J Med Internet Res. 2023 Jun 14;25:e47672. doi: 10.2196/47672 (PMC10337458; doi:10.2196/47672)
Supplement: Multimedia Appendix 4 [file jmir_v25i1e47672_app4.docx]

**MULTIMEDIA APPENDIX 4: Figure S1. Flow Diagram Health Circuit**

**CONSORT 2010 Flow Diagram Health Circuit**

Allocated to control group (n=18)

- Received allocated intervention (n=18)
- Did not received allocated intervention (n= 0)

Allocated to intervention group (n=41)

- Received allocated intervention (n=39)
- Did not received allocated intervention (n= 2)
  - Technological problems (n = 2)

## Allocation

Excluded (n=41)

- Died (n= 5)
- Did not answer telephone (n=5)
- Not meeting inclusion criteria(n=20)
- Refuse (n= 11)

Center randomized

Lost to follow-up (n=2)

- Died (n=2)

Total (n=16)
♦ Excluded from analysis (n=0)

## Follow-Up

Lost to follow-up (give reasons) (n=8)

• Technological problems (n = 2)

• Died (n = 1)

• Worsening of health (n = 2)

• Lost mobile device (n = 1)

• Unreachable (n = 2)

## Analysis

Total (n=31)
♦ Excluded from analysis (n=0)

## Enrollment

Assessed for eligibility (n=100)
