# Supplementary material for: A Practice-Proven Adaptive Case Management Approach for Innovative Health Care Services (Health Circuit): Cluster Randomized Clinical Pilot and Descriptive Observational Study
Source: J Med Internet Res. 2023 Jun 14;25:e47672. doi: 10.2196/47672 (PMC10337458; doi:10.2196/47672)
Supplement: Multimedia Appendix 5 [file jmir_v25i1e47672_app5.docx]

**MULTIMEDIA APPENDIX 5:** Digital baseline characteristics in the intervention group and baseline use of technologies and health information sources

**Table S1**. Digital baseline characteristics in the intervention group.


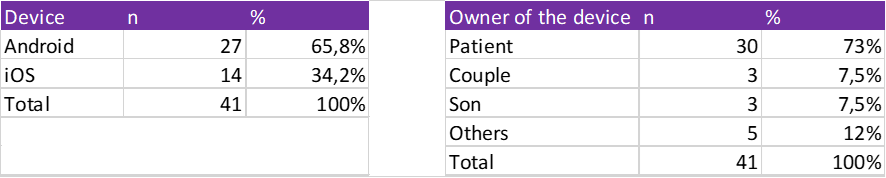


**Figure S2.** Baseline use of technologies and health information sources.
